# Report of a Case of Pyosalpinx Relieved by Drainage through the Vagina

**Published:** 1893-04

**Authors:** Jeff S. Davis

**Affiliations:** Montevallo, Ala.


### REPORT OF A CASE OF PYOSALPINX RELIEVED BY DRAINAGE THROUGH THE VAGINA.

By JEFF S. DAVIS, M. D.,
Montevallo, Ala.

Several months ago Dr. Paul F. Munde contributed a paper to the *American Journal of Obstetrics* on The Conservative Treatment of Salpingitis, in which he advocated draining the Fallopian tubes through the vagina for the relief of a certain class of cases of pyosalpinx ; *i. e.*, where only one tube is diseased, and that adherent.

A short while after reading his article I had an opportunity to test the efficacy of this method of treatment, and the results attained are so promising I am of the opinion that the cases will bear reporting. The technique is very simple, and consequently the operation will specially interest the general practitioner. The possession of an anatomical knowledge of the parts with some dexterity in manipulation are the only essential elements in its performance.

A negro woman, thirty-two years of age, the mother of four children, youngest eighteen months old, consulted me for recurring attacks of excruciating pain in right pelvic region attended by fever and disturbances of the gastro-intestinal tract. She suffered almost incessantly from a heavy dragging sensation in pelvis and lower part of abdomen, but was able to be up, except during the severe attacks, which came on about every six weeks, lasting from four to six days. She was very much emaciated and presented every appearance of extreme nervous prostration. She was attended by a midwife in her last confinement and was thirty-six hours in labor. Five months after this labor she had her first attack but did not remember when she first commenced to suffer, probably very soon after confinement. Had not menstruated since last pregnancy. A vaginal examination revealed a fluctuating, immovable mass, the size of a small sausage in the right pelvic region. The uterus was only very slighly movable. My father, Dr. Ralph Davis, saw the case with me, and on exploration aspiration was decided upon. The woman being very nervous was anæsthetized, and the needle of a small aspirator was pushed through the vaginal wall into the mass. A small quantity of dark and quite offensive pus was thus obtained. At the point at which the needle was inserted a larger and longer needle was introduced and attached to Allen's surgical pump, with which we were enabled to aspirate four ounces of pus. The tube was thoroughly washed out before withdrawing the needle. The opening was then enlarged and a small rubber drainage tube applied which was retained in position by means of a small silver safety pin passed through the mucous membrane of the vagina and each wall of the rubber tube. The tube was three inches long. Iodoform gauze was loosely packed around it to afford support and prevent any weight at the attached extremity. The vault of the vagina was painted with equal quantities of iodine and glycerine as recommended by Dr. Munde. Iron, cod liver oil and lactopeptine constituted the general treatment, and opiates were given when necessary to control pain or secure sleep. The iodoform gauze was removed daily and a hot vaginal douche administered ; the tube was washed out with Allen's pump every third day. Under this treatment she gradually improved. At the end of six weeks the pelvic pain had been greatly alleviated and there was a very noticeable difference in the size of the tube. The case thus progressed very favorably until the latter part of the eighth week, when she was suddenly prostrated with one of the severe attacks which she informed me was the worst that she had ever had. I was very much discouraged and on the verge of pronouncing the treatment a failure.

She was quieted with opiates and on the day following a thorough vaginal examination was made. The lower part of the tube was found very much reduced in size, and in it I could discover no fluctuation, but higher up a distinct enlargement with very marked fluctuation was detected. With much difficulty I succeeded in introducing a similar tube to the first at that point and was rewarded by seeing quite a quantity of pus pass out. The tube was evidently divided by an adhesive partition and the first tube had not drained the upper chamber. From this time her improvement was more pronounced and the tube gradually diminished in size. At the expiration of three months, during which time the drainage was continued, no enlargement of the tube could be detected and her general health was very good. The menstrual functions have not been established though there is no evidence of disease in the left tube or ovary.

I am not at present willing to assert that she is permanently *cured*, but there is certainly no evidence of inflammatory trouble about the uterine appendages, and her general health is constantly improving.

From this report we see that to obtain any degree of success by this method patience and perseverance on the part of the physician are absolutely essential. Relief will always come slowly, the patient often becoming disheartened and the surgeon discouraged. Owing to these objectionable features of the method I would in a case of this kind, with suitable environments—all the facilities for good nursing and antiseptic precautions at hand—advise the removal of the appendage. By this method relief is sooner obtained, is more certain, and the body is forever rid of a diseased and functionless organ. On the other hand where the patient's life would be jeopardized by careless, inefficient attention and bad hygeinic surroundings, or the operator fears to test his knowledge or skill in opening the cavity, and is unable to procure proficient aid, the method adopted in the case reported should be resorted to in those cases to which it is adaptable and it will finally effect a cure.

